# Germinal proto-mitochondria from rat liver

**DOI:** 10.1016/j.bbrep.2019.100710

**Published:** 2019-11-23

**Authors:** Nikolai Vekshin, Vladimir Kovalev, Alina Chaplygina

**Affiliations:** Institute of Cell Biophysics of RAS, 142290, Pushchino, Moscow Region, Russian Federation

**Keywords:** Proto-mitochondria, Mitochondria, Post-mitochondria, Mitochondrial DNA, Flavoproteins, Respiratory chain, Lipofuscin, MC, mitochondria, PRMC, protomitochondria

## Abstract

A number of properties of the smallest (less than 0.2 μm) germinal proto-mitochondria (PRMC) from rat liver have been studied. These PRMC were obtained by filtering the light fraction of hepatic mitochondria (MC) through calibrated millipore membranes. Germinal PRMC contain in general the same proteins as MC. However, they have the reduced content of flavoproteins and zero cytochrome oxidase. Germinal PRMC, in contrast to MC, almost does not contain the “aging pigment” - lipofuscin. They have DNA; the DNA/protein ratio in them is much higher than in MC, i.e. they are poor in protein. The obtained results support the earlier assumption that MC in specialized animal cells can arise from germinal PRMC - particles smaller than 0.2 μm containing DNA. It is assumed that the DNA molecules enter to cytoplasm during degradation of old MC serves as a seed for the formation of PRMC (with the connection of nuclear DNA).

## 1 Introduction

Mitochondria (MC) are two-membrane organelles of eukaryotic aerobic cells, one of the main functions of which is the generation of the transmembrane potential, accompanied by the synthesis of ATP [1]. MC of different cells vary in size, density, participation in metabolism, aging and apoptosis [[2], [3], [4], [5]]. In each animal cell, there are usually many hundreds and thousands of MC. In specialized cells of the organs (liver, muscles, heart, kidneys, etc.), MC practically do not divide, but arise presumably in the form of germinal proto-MC (PRMC) with a diameter of less than 0.2 μm, which gradually grow to mature MC of about 1 μm, with time degrading to the old post-MC of 1–6 μm in diameter [6,7]. The ratio of PRMC, MC and post-MC populations in each cell depends on the cell type, age of the animal and other parameters (6). PRMC of less than 0.45 μm in specialized animal cells are about 30% of all MC [6,7].

PRMC were first isolated in our laboratory from the cells of the organs of animals (heart and liver) and some of their properties were studied [[7], [8], [9], [10], [11], [12], [13]]. Using hepatic PRMC with a diameter less than 0.45 μm, it was shown that these spherical particles are not MC fragments. They respire and exhibit some phosphorylating activity. PRMC with a diameter of 0.25–0.45 μm have a complete respiratory chain, as evidenced by oxygen consumption on cytochrome oxidase during the oxidation of succinate and NADH. In addition, PRMC have high succinate- and NADH-tetrazolium reductase activities. Horizontal electrophoresis showed that PRMC of 0.25–0.45 μm contain many DNA copies of 16 kilo-bases (in MC, in addition to 16-kilo-base, there are minor satellite DNA). Gel filtration and vertical electrophoresis showed the coincidence of most protein bands of the 0.25–0.45 PRMC and MC, but with some quantitative differences. The protein tryptophan fluorescence spectrum of PRMC (0.25–0.45) is almost identical to MC, but the tyrosine component is slightly increased. In addition, using electron microscopy, it was determined that the 0.45 μm PRMC possess both outer and inner membranes.

The purpose of this work is to study a number of properties of small germinal “embryonic” PRMC (from rat liver) with a diameter of less than 0.22 μm. This is necessary to understand the mechanism of their occurrence and maturation to full-scale MC in specialized animal cells.

## 2 Materials and methods

### 2.1 Isolation

The total fraction of MC from the liver of Wistar rats (contained in standard conditions) was isolated according to the usual procedure with some modifications [[7], [8], [9]], which allow obtaining a wide range of large heavy and small light organelles. The work was carried out at 4 °C, all solutions were cooled on ice. The liver of a 4-month-old rat was placed in 60 ml of cold isolation medium, containing 250 mM sucrose, 1 mM EDTA and 10 mM HEPES (pH 7.5), and then the liver was extruded through a press and homogenized in a glass homogenizer with a teflon pestle for cell destruction. The homogenate was centrifuged for 10 min on a PC-6 centrifuge (“Dastan”) at 1000 rpm to precipitate the red blood cells and un-damaged hepatocytes. The precipitate was discarded and the supernatant was centrifuged for 15 min at 3300 rpm to obtain a precipitate of MC (heavy fraction) which, after re-suspension, was diluted with the isolation medium and packed into eppendorf tubes. The remaining supernatant was centrifuged at 5600 rpm for 20 min. The precipitate (light fraction) was suspended in 6 ml of the same medium and immediately passed through nitrocellulose millipore Whatman's membranes with a pore size of 0.2 μm or millipore filters (Italy) of 0.22 μm. The resulting filtrate of PRMC was divided into aliquots and used for experiments. Before spectroscopic experiments, 0.5% SDS was added to dissolve the particles and minimize light scattering. For comparison, a heavy fraction of MC or calibrated MC was used, which was obtained by filtering a dilute suspension of heavy MC through 1-μm filters, collecting the MC residues from these filters by soaking in 0.5% SDS.

### 2.2 Scattering

The scattering of light on each individual particle was eliminated by destroying the PRMC and MC by means of SDS, which converted muddy suspensions into completely transparent solutions. The residual contribution of light scattering in the region of absorption bands in the UV and visible regions was removed by approximation from 700 nm, where there is no absorption, but only light scattering.

### 2.3 Absorption

Absorption spectra in the UV region were recorded on a Carry Eclipse (USA) or M-40 spectrophotometer (Zeiss, Germany) in a compartment for turbid samples, which allows compensating residual light scattering with a hemispherical detector.

### 2.4 Fluorescence

Fluorescence spectra were recorded in 1-cm quartz cells with MF44B spectrophotometers (PerkinElmer, USA) and Carry Eclipse (Agilent Technologies, USA) using practically transparent detergent solutions obtained by treating the PRMC and MC with 0.5% SDS (without potassium ions, to minimize aggregation of the detergent).

### 2.5 Protein content

The total protein content of PRMC and MC in spectroscopic experiments was usually 0.35 mg/ml. The protein concentration was determined by the UV method [14].

### 2.6 Optical microscopy

Optical microscopy of PRMC and MC was carried out on LUMAM I-2 (LOMO) and Axio Imager.Z1 (Carl Zeiss) microscopes in the transmitted, phase-contrast and fluorescent mode. For staining of PRMC and MC, a fluorescent dye was used - Hoechst 33342, which specifically binds to DNA [10].

### 2.7 Infrared absorption

The IR absorption spectra were recorded on infrared Fourier spectrometer FT-801 (Simeks, RF) to obtain films from PRMC and MC (isolated from rat liver in the presence of 300 mM sodium chloride without sucrose, EDTA and HEPES, which absorb in the IR region) by drying them on calcium fluoride plates.

### 2.8 Electrophoresis

Protein composition of PRMC and MC was compared by the method of denaturing SDS gel electrophoresis in 10% polyacrylamide gel. A sample 25 μl of PRMC or MC of a protein concentration 100 μg was applied to the lane. Protein Markers (New England Biolabs, Ink.) were used as protein standards.

### 2.9 Respiration

Respiratory activity of PRMC and MC was determined from the rate of oxygen consumption with the help of Clark's electrode “Expert-001" (LLC “Ekoniks”, RF). The incubation medium contained 5 mM succinate, 2 mM potassium phosphate and 150 mM sucrose (pH 7.5).

## 3 Results and discussion

**Fig. 1:**
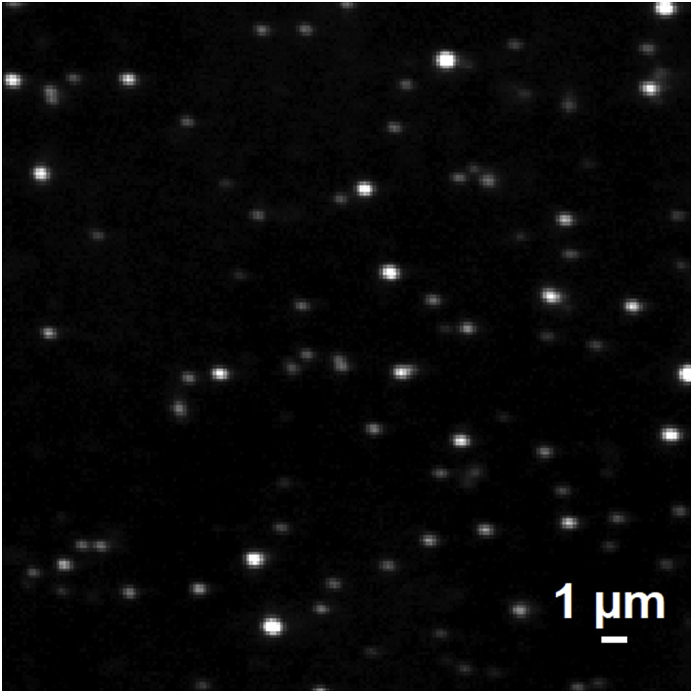
Optical microscopy of PRMC from rat liver obtained by filtration of the light fraction of MC through a millipore filter with a pore diameter of 0.2 μm, staining with Hoechst, in the regime of “fluorescence” (excitation - 350 nm; interference filter). Microscope Axio Imager.Z1, the lens EC Plan-Neofluar 100x/1.30 Oil Iris M27.

PRMC <0.2 μm are not visible in optical microscopy either in transmitted light, in phase-contrast mode, or in fluorescent mode (not shown in figures). This is due to facts that a) the particle size is much smaller than the wavelength of the light wave, b) refractive index of the particles is not high, c) optical density of each particle is negligible. However, when staining with Hoechst (specific dye for DNA), they become visible in phase-contrast and fluorescent regimes (Fig. 1). Moreover, in the fluorescent mode, the image is much clearer than in the phase-contrast mode. This is due to the fact that the fluorescent mode uses shorter wavelength lighting - 350 nm. The diameter of some particles in Fig. 1 seems larger than 0.2 μm because they are out of focus, and also because of diffraction and dispersion. Since Hoechst is able to specifically stain the DNA, the staining means that the PRMC <0.2 μm contain a noticeable amount of DNA. This result agrees with our data of horizontal electrophoresis (11) on the presence of DNA in the PRMC with a diameter of 0.25–0.45 μm.

**Fig. 2:**
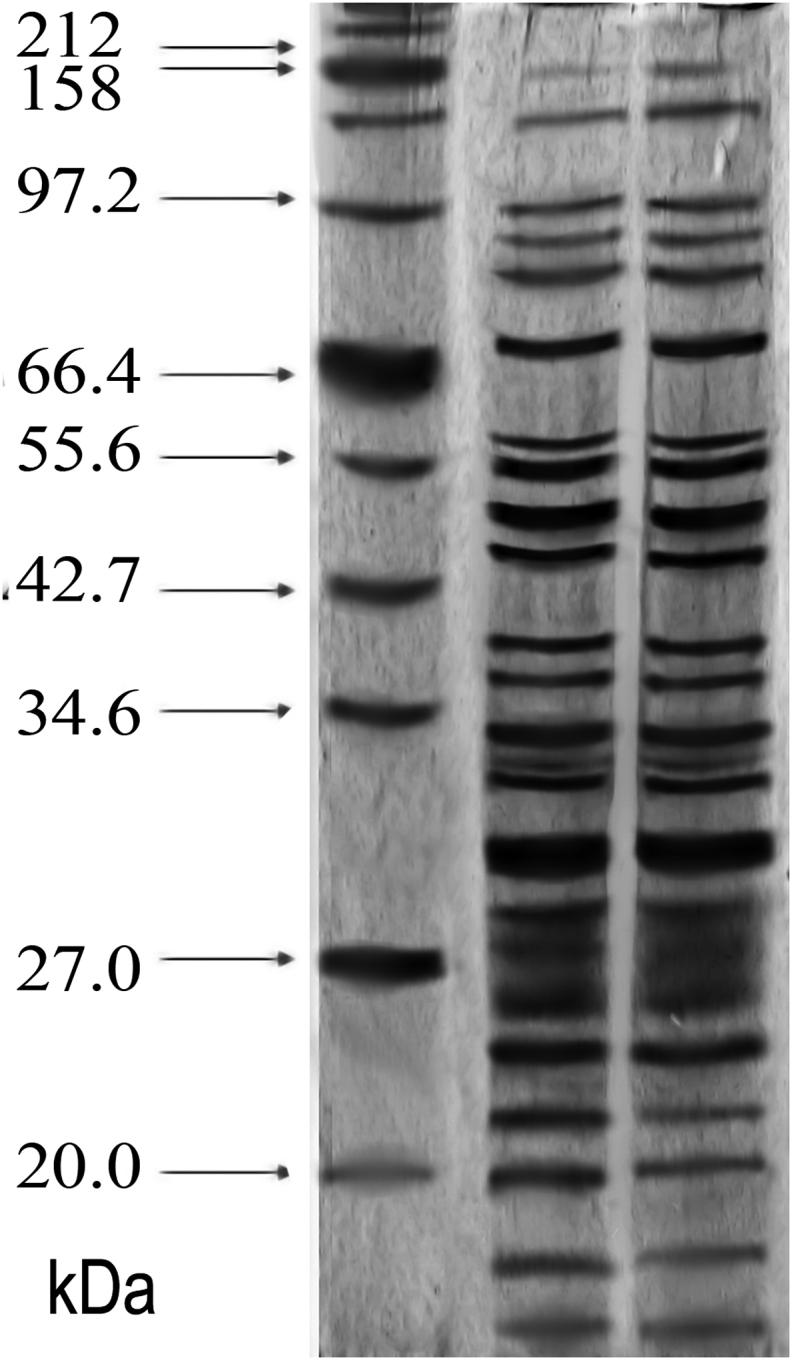
Electrophoresis of proteins of PRMC <0.22 μm (right) and MC (center) in 10% polyacrylamide gel in the presence of 0.1% SDS, versus protein standards (left).

A comparative analysis of the total protein composition of PRMC and MC by gel electrophoresis showed no significant differences in the major proteins of the germinal PRMC and MC (Fig. 2), which proves the complete relationship of these organelles. The quantitative difference exists only for low-molecular proteins ~20 kDa, the content of which in PRMC is lower than in MC. It also follows from electrophoresis that the fraction of germinal PRMC, if it contains any protein impurities, does not differ appreciably from the impurities in the MC fraction.

**Fig. 3:**
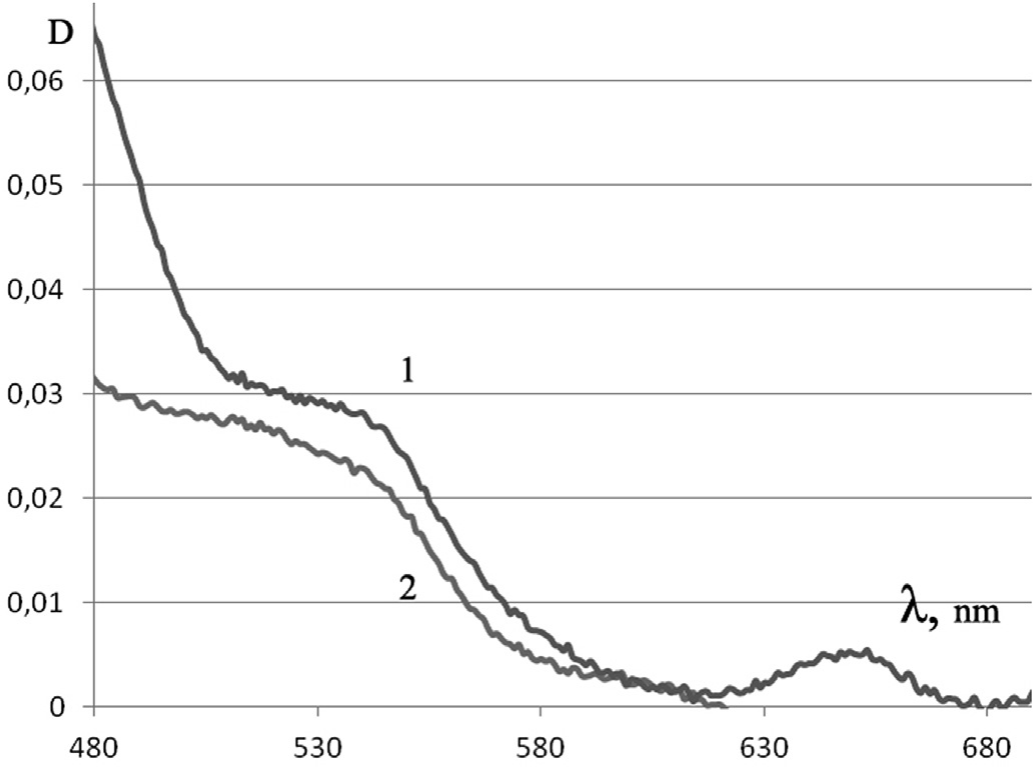
Absorption spectra of cytochromes of MC (1) and PRMC <0.2 μm (2) after destruction of the suspension particles by detergent (0.5% SDS). Characteristic spectra are shown, minus light scattering.

In terms of minor proteins, however, the differences between PRMC and MC are quite significant. Judging by the characteristic absorption spectrum in the visible region, the PRMC <0.2 μm have fewer cytochromes than MC (weakened Soret band at 480 nm, the long-wave part of the band - Fig. 3), and they do not have cytochrome oxidase at all (at 650 nm, Fig. 3). Thus, PRMC <0.2 μm do not have formed respiratory centers. That is why, in contrast to the PRMC with a diameter of 0.25–0.45 μm, which have respiration and even respiratory control [7,11], PRMC < 0.2 μm practically do not respire (data on oxygen consumption are not given).

**Fig. 4:**
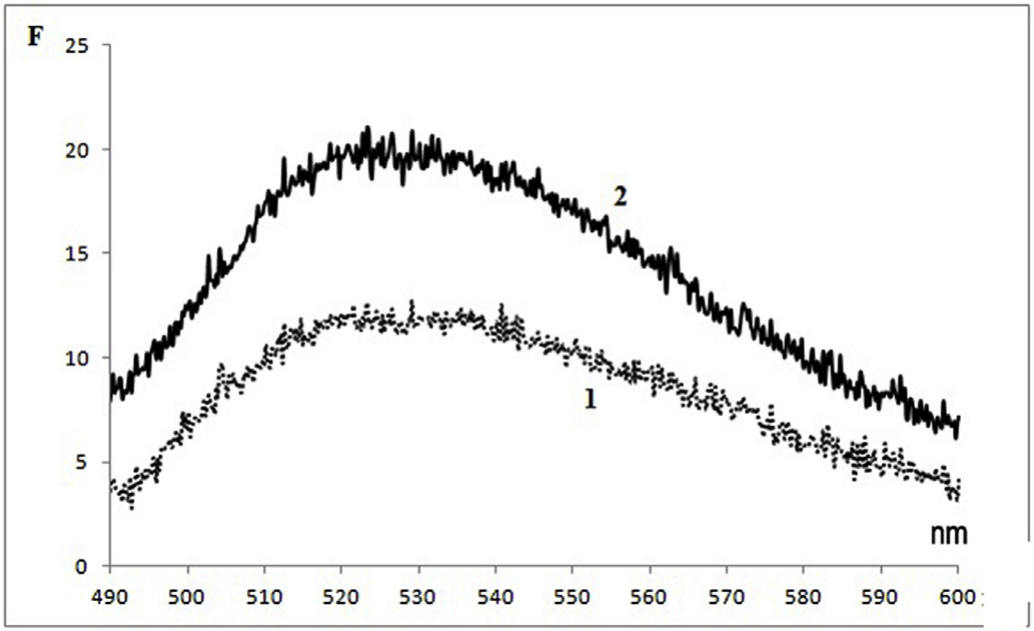
Flavin fluorescence spectra (excitation - 450 nm) of PRMC <0.2 μm (1) and MC (2) after destruction of the suspension particles by a detergent (0.5% SDS).

Judging by the intensity of the flavin fluorescence (maximum at 525 nm), the total content of flavoproteins in the germinal PRMC is 35% (±8%) less on average than in MC (Fig. 4). This is quite an expected result, if we assume that the ripening of germinal PRMC requires the flavoproteins to increase in order to ensure the functioning of the respiratory chain. In hepatic MC, there are FMN-containing NADH dehydrogenase and several FAD-containing flavoproteins [15]. It can be concluded that there are fewer of them in germinal PRMC than in MC.

**Fig. 5:**
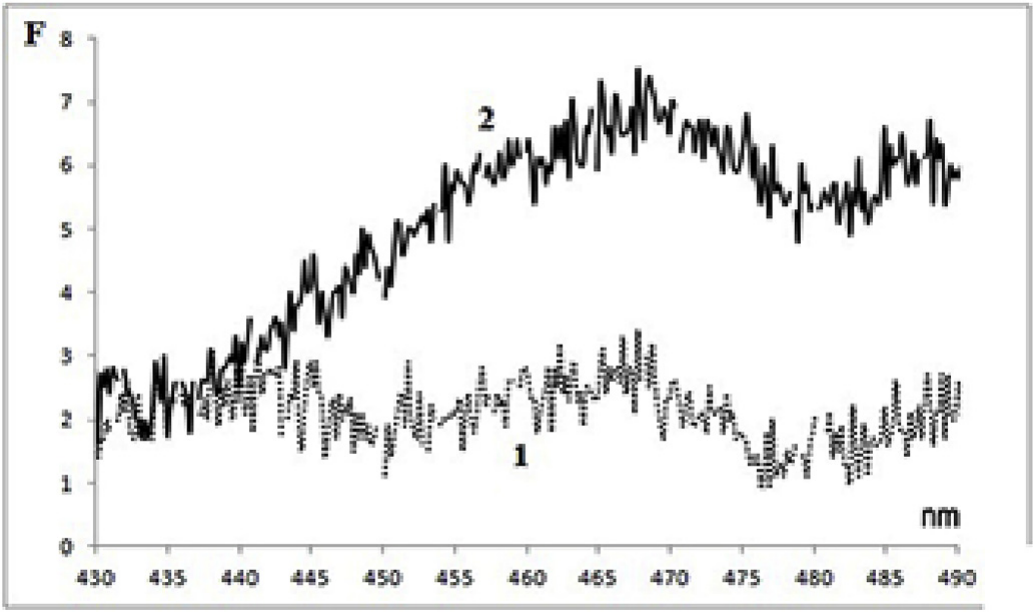
Spectral characteristic of lipofuscin fluorescence (excitation at 360 nm) of PRMC <0.2 μm (1) and MC (2) after destruction of the suspension particles by detergent (0.5% SDS). The intensity at 490 nm belongs to the contribution from the flavin fluorescence band.

The germinal PRMC practically do not contain the “aging pigment” - lipofuscin: there is no characteristic “blue” band in the fluorescence spectrum at the 465 nm region, while it is quite noticeable in MC (Fig. 5). In different experiments on various adult rats, the intensity of the lipofuscin band in these PRMC was 4–5 times lower than in MC. This important fact means that PRMC <0.2 μm are very young organelles, in which, unlike MC, the Schiff bases have not yet formed (lipid peroxidation and protein-protein cross-links are negligible).

Interestingly, in the monograph [6], a strong lipofuscin fluorescence of post-MC was described. We have shown [12] that lipofuscin is formed in aging MC without the involvement of lysosomes, and the appearance of lipofuscin can be activated by moderate heating. It should be noted that the band at 465 nm under our conditions does not belong to NADH or NADPH, since MC were initially incubated before the complete oxidation of endogenous substrates.

**Fig. 6:**
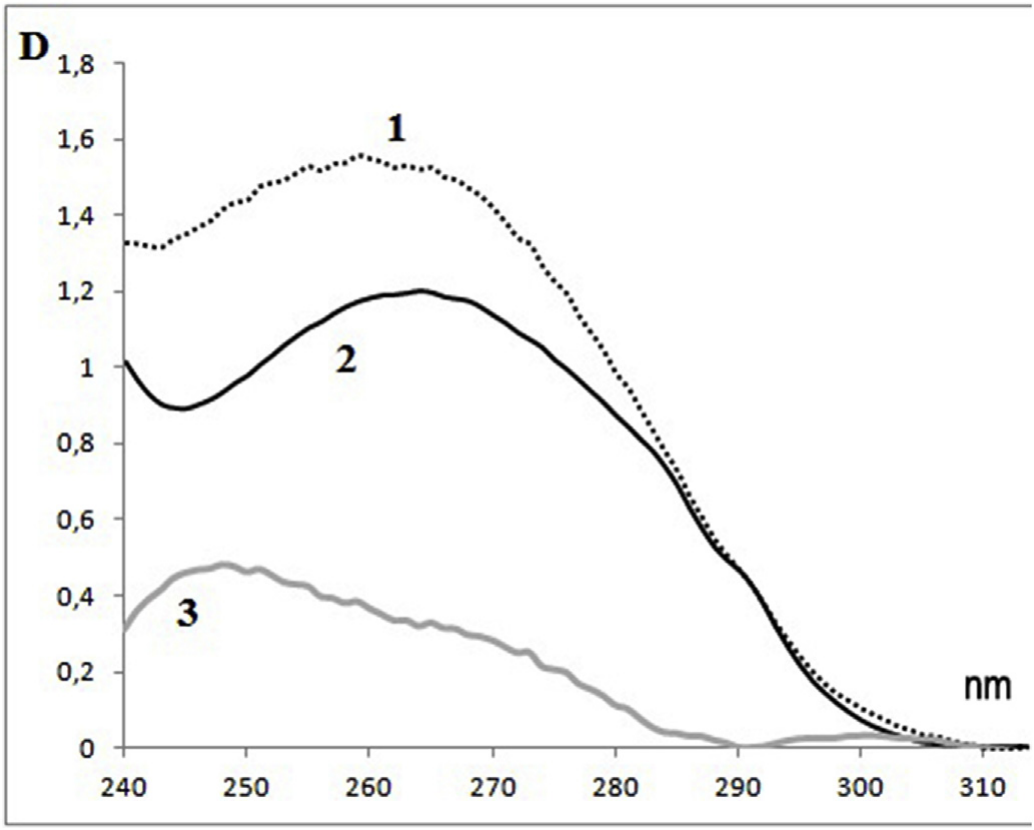
UV absorption spectra of PRMC <0.2 μ (1) and MC (2) after destruction of the suspension particles by a detergent (0.5% SDS), as well as the difference spectrum (3). Characteristic spectra are shown, minus the residual light scattering.

The UV absorption spectrum of germinal PRMC shows more DNA than protein: the maximum lies at 258 nm - in the shorter-wave region than the MC spectrum, where the contribution of DNA to UV absorption is also significant (Fig. 6). A small tryptophan arm of proteins clearly appears in the spectrum at 288 nm. The difference spectrum of UV absorption of PRMC and MC roughly corresponds to the DNA band. Germinal PRMC have less total protein than MC, and therefore - a greater spectral ratio of DNA/protein. It should be emphasized that the presence of a clearly pronounced DNA absorption band in the PRMC spectrum once again confirms that the fraction of germinal PRMC is not too contaminated with microsomes or other particles. Otherwise, the spectrum would not be mainly DNA, but would be protein-like - with the main maximum at 280 nm.

**Fig. 7:**
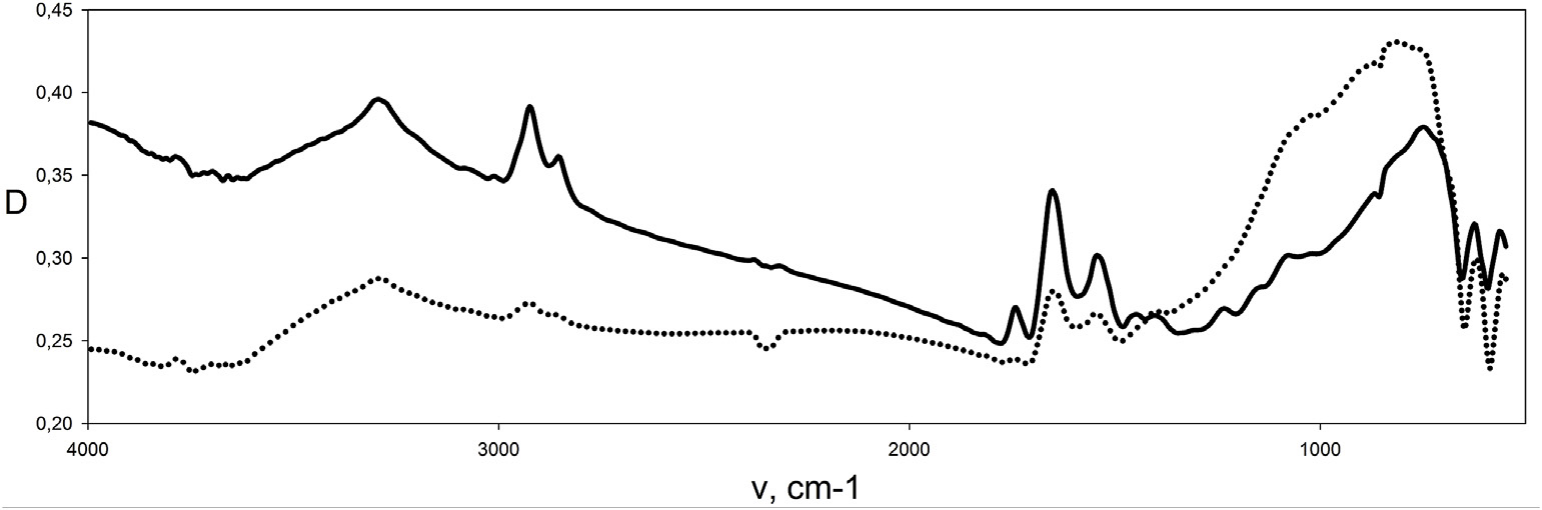
IR absorption spectra of the PRMC <0.22 μ (···) and MC (−).

When comparing the absorption spectra of the PRMC <0.2 μ and MC in the middle IR region (Fig. 7) with the IR spectra of marker proteins (BSA, etc.), pure DNA and lipids (their spectra are not shown here), it was found that the main contribution to the IR absorption of PRMC and MC, as expected, is produced by proteins. A set of intense bands at 3293, 2924, 1652 and 1542 cm-1 in MC and PRMC belongs to them. A small band at 3786 cm-1 belongs to purines and pyrimidines of DNA [16]. The doublet 640 - 610 cm-1 belongs to the DNA phosphates: a similar doublet is present in the spectrum of sodium phosphate (the spectrum is not given). A wide band with maxima in the region of 800-750 cm-1 belongs to lipids and other components.

The peak at 3293 cm-1 is due to stretching vibrations of NH- and OH-groups, and the peak at 2924 cm-1 is due to stretching vibrations of OH and CH groups [16]. The peak at 1652 cm-1 is due to stretching vibrations of CO-groups, as well as deformation vibrations of OH-groups of bound water left after drying of PRMC and MC. The peak at 1542 cm-1 is due to the deformation vibrations of the NH-groups.

The IR spectrum of germinal PRMC <0.2 μm is very similar to the MC spectrum, which again confirms the close relationship of PRMC and MC. However, the intensities of the bands are significantly different. The position of the peaks does not coincide completely everywhere. Protein bands in PRMC are weaker than in MC, and lipids are stronger on the contrary. Apparently, PRMC <0.2 μm contain less protein, but more lipid.

The significant difference between MC and PRMC in the intensity of a broad structure-less band in the range from 4000 cm-1 to 2000 cm-1 is due to the presence of more bound water in the MC, as well as to large light scattering. Therefore, after calculating the intensities of protein bands, the contribution of this structure-less band was subtracted.

Judging by the intensities of protein and nucleotide IR bands, the protein/DNA ratio in PRMC is lower than in MC, i.e. the germinal PRMC have lower amount of total protein.

**Fig. 8:**
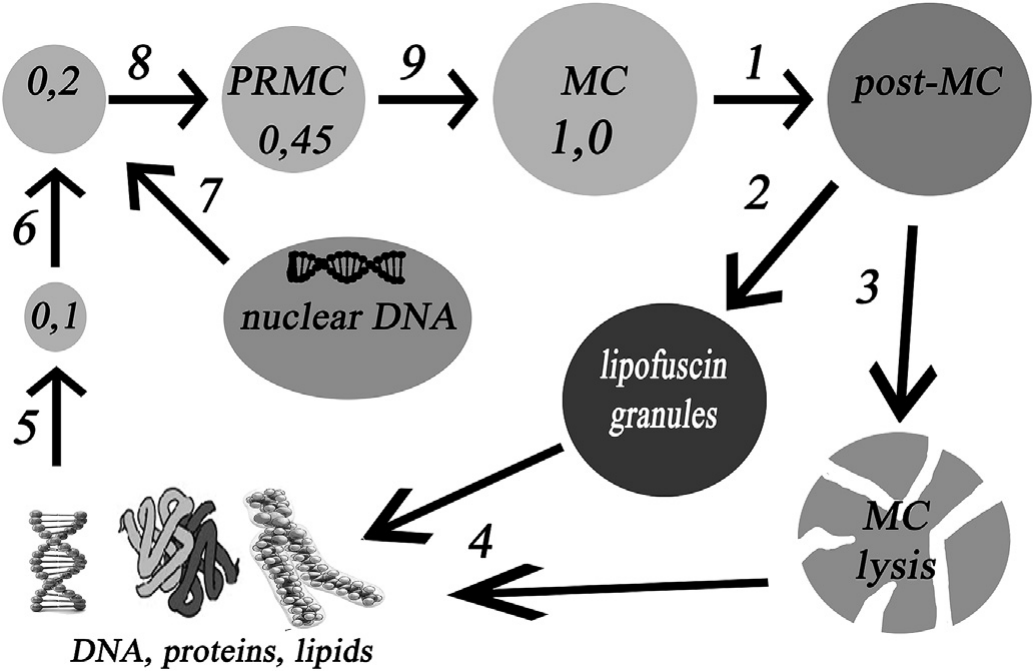
Scheme of the MC life cycle in specialized animal cells: 1 - aging of MC to post-MC, 2 - post-MC degradation to lipofuscin granules or 3- lysis of post-MC by proteases and lysosomes, 4 - DNA release (with some proteins) into cytoplasm, 5 - nucleation of a ~0.1-μm PRMC, 6 - synthesis of a number of membrane proteins, 7 - cooperation with nuclear DNA, synthesis of the majority of proteins and membrane formation, 8 - growth of PRMC, 9 - further growth and development of PRMC.

Based on the data obtained, it is possible to propose a scheme (Fig. 8) of the life cycle of MC in specialized cells of animals. Small germinal PRMC of about 0.1 μm in size arise from DNA emerging into the cytoplasm from post-MC or degraded MC. To synthesize membrane proteins of PRMC, the nuclear DNA has to be connected (it is not yet known what exactly is the signal for activation of the necessary genes of nuclear DNA). Damaged, oxidized or mutated DNA that is incapable of forming germinal PRMC is likely to be cleaved in the cytoplasm by DNA-ase and nucleases. In the course of the life cycle of the cell, PRMC germination occurs, followed by their growth (0.2–0.45 μm), and then - growth and development into adult MC. Then, MC aging to post-MX takes place with accumulation of large amounts of lipofuscin, up to the formation of lipofuscin granules. If the proteases and lysosomes are sufficiently active, then the lysis of old MC occurs with the release of DNA from them into the cytoplasm.

The results obtained for the PRMC <0.22 μ are in good agreement with the results for the PRMC <0.45 μm [11] and confirm the earlier assumption that MC in specialized animal cells mainly arise not by fission or budding, but from germinal PRMC - particles smaller than 0.2 μm, containing DNA and a number of proteins.

Judging from the data of optical microscopy and filtration, the amount of germinal PRMC <0.2 μm in the hepatic cells does not exceed 10% of the number of mature MC. This agrees with the data of optical dark-field microscopy of cells (6) and suspensions of different fractions of MC [17].
